# Proximity-Induced Superconductivity in a 2D Kondo Lattice of an *f*-Electron-Based Surface Alloy

**DOI:** 10.1021/acs.nanolett.4c04796

**Published:** 2024-10-25

**Authors:** Howon Kim, Dirk K. Morr, Roland Wiesendanger

**Affiliations:** † Department of Physics, University of Hamburg, D-20355 Hamburg, Germany; ‡ Department of Physics, University of Illinois at Chicago, Chicago, Illinois 60607, United States

**Keywords:** superconductor-magnet hybrid system, surface alloy, Kondo lattice, superconductor, hybridization gap, Yu-Shiba-Rusinov band

## Abstract

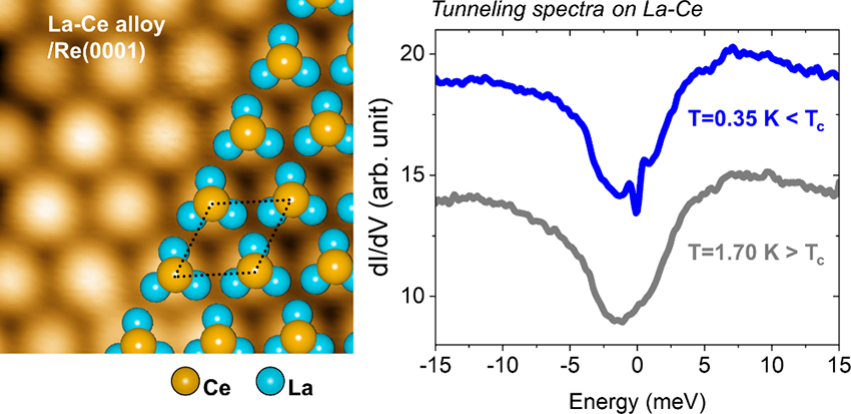

Realizing hybrids of low-dimensional Kondo lattices and superconducting substrates leads to fascinating platforms for studying the exciting physics of strongly correlated electron systems with induced superconducting pairing. Here, we report a scanning tunneling microscopy and spectroscopy study of a new type of two-dimensional (2D) La–Ce alloy grown epitaxially on a superconducting Re(0001) substrate. We observe the characteristic spectroscopic signature of a hybridization gap evidencing the coherent spin screening in the 2D Kondo lattice realized by the ultrathin La–Ce alloy film on normal conducting Re(0001). Upon lowering the temperature below the critical temperature of rhenium, a superconducting gap is induced exhibiting an energy asymmetry of the coherence peaks that arises from the interaction of residual unscreened magnetic moments with the superconducting substrate. A positive correlation between the Kondo hybridization gap and the asymmetry of the coherence peaks is found.

Strongly correlated material systems have been an extremely active and exciting research area in condensed matter physics over the past decades.^1,2^ Outstanding examples include heavy-Fermion compounds^3^ with extremely large effective electron masses exhibiting either superconductivity or magnetic order,^4^ high-*T*_*c*_ cuprates,^5^ Kondo lattice systems,^6^ Kondo insulators,^7^ to mention only a few. Fascinating properties of strongly correlated electron systems range from unconventional superconductivity^8^ to non-Fermi-liquid behavior, in particular close to quantum critical points.^9^ Many of the unconventional superconductors are low-dimensional, layered materials close to a quantum phase transition. The interplay between different fundamental interactions results in complex phase diagrams and the emergence of novel exotic states of matter.

Most of the early pioneering work on strongly correlated electron systems has been performed based on bulk materials. The quality of the single crystals proved to be of great importance for revealing the intrinsic exciting physical properties of strongly correlated materials including cuprates and *f*-electron compounds.^4^ Major challenges have been the purification of the starting elements as well as the microscopic material characterization, apart from bulk-sensitive methods such as X-ray diffraction. More recently, within the past two decades, investigations have focused more on low-dimensional systems, including ultrathin films,^10−14^ artificial 2D atomic arrays,^15^ and quasi-1D chains^16,17^ revealing Kondo physics.^18,19^

Remarkably, almost all previous investigations of atomic-scale Kondo systems using scanning tunneling microscopy and spectroscopy (STM/STS) techniques have been performed with transition metal-based magnetic impurities where the localized magnetic moments are due to *d*-electrons.^20^ An exception is the early work on the Ce on Ag(111) system,^21^ where the authors assumed to study isolated Ce adatoms on the Ag substrate, but which later turned out to be Ce clusters.^22^ Based on this particular sample system, namely Ce on Ag(111), it has been demonstrated that ordered 2D arrays of Ce adatoms can be achieved by self-assembly.^23−25^ However, at the measurement temperature of around 4 K, neither a Kondo state of the individual Ce adatoms, nor the transition to the behavior of a 2D Kondo lattice could be observed.

Here, we report on the successful preparation of an ultrathin La–Ce alloy film on a clean Re(0001) substrate under ultrahigh vacuum conditions. The choice of the La–Ce combination was motivated by the fact that the LaCe bulk material is a well-known Kondo alloy with a Kondo temperature below 1 K, which was already investigated intensively in the early seventies of the previous century.^26,27^ The atomic-scale structure of the new type of 2D La–Ce alloy has been investigated by atomic-resolution STM, whereas the spatially resolved electronic properties have been revealed by STS measurements above and below the superconducting transition temperature (*T*_*c*,*Re*_∼ 1.6 K) of the Re(0001) substrate.

Tunneling spectroscopic data of the 2D La–Ce alloy on normal-conducting Re(0001) reveal a robust asymmetric double-peak resonance at the Fermi level, which is a hallmark of a Kondo lattice revealing a Kondo hybridization gap.^28−36^ Below the superconducting transition temperature of the Re(0001) substrate, superconducting pairing is induced in the 2D La–Ce alloy via the proximity effect, resulting in the opening of a gap and the formation of coherence peaks. Surprisingly, the coherence peaks are located at asymmetric energies, *E*_*c*,2_ ≠ – *E*_*c*,1_, apparently breaking the particle-hole symmetry of the superconducting state. Based on model calculations, we show that this energy asymmetry arises from the presence of residual unscreened magnetic moments, which is revealed by a spin-polarized STM tip and leads to the formation of a Yu-Shiba-Rusinov (YSR) band.^37−40^ We find that the formation of the YSR band is suppressed with increasing size of the Kondo hybridization gap, leading to a uniform shift of the coherence peaks to higher energies. The new type of 2D Kondo lattice proximitized to a superconductor provides novel insight into the competition between Kondo screening and superconductivity and offers an exciting route toward the artificial design of low-dimensional superconducting heavy-Fermion systems.^41,42^

Figure 1(a) shows a representative STM topographic image of the ultrathin La–Ce alloy film grown on Re(0001). Ultrathin La–Ce alloy films were prepared by a two-step *in situ* process under UHV conditions. Details of the epitaxial thin-film growth are described in the Methods section. The 2D La–Ce alloy starts to form at step edges of the pristine Re(0001) surface while parts of the Re(0001) terraces are covered with Ce clusters without a La wetting layer underneath. The 2D La–Ce alloy layer exhibits an ordered atomic lattice structure together with some randomly distributed local defects, being primarily Ce vacancy sites. A high-resolution zoomed-in STM image in Figure 1(b) clearly shows the hexagonal lattice structure of the La–Ce alloy layer with a lattice constant of about 0.71 nm, which is equivalent to √7 *a*_*Re*_, where *a*_*Re*_ is the lattice constant of the Re(0001) substrate (*a*_*Re*_ = 0.274 nm). Interestingly, above the Ce vacancy sites of the La–Ce lattice, trimer-like structures formed by La atoms are visible in STM images obtained at low sample bias voltage [see Supplementary Note 1 for the atomic structure model of the La–Ce alloy film]. Based on the atomic-resolution STM data, an atomic structure model of the 2D La–Ce alloy can be derived, which is overlaid on the STM image of Figure 1(b). Based on this model, the 2D La–Ce layer is composed of an ordered hexagonal array of La_3_Ce complexes. Intermixing of lanthanides with the chosen Re(0001) substrate does not occur even during high-temperature annealing, in strong contrast to lanthanide (La, Ce, Gd) - based surface alloys formed on noble metal substrates, such as Au(111).^43,44^

**Figure 1:**
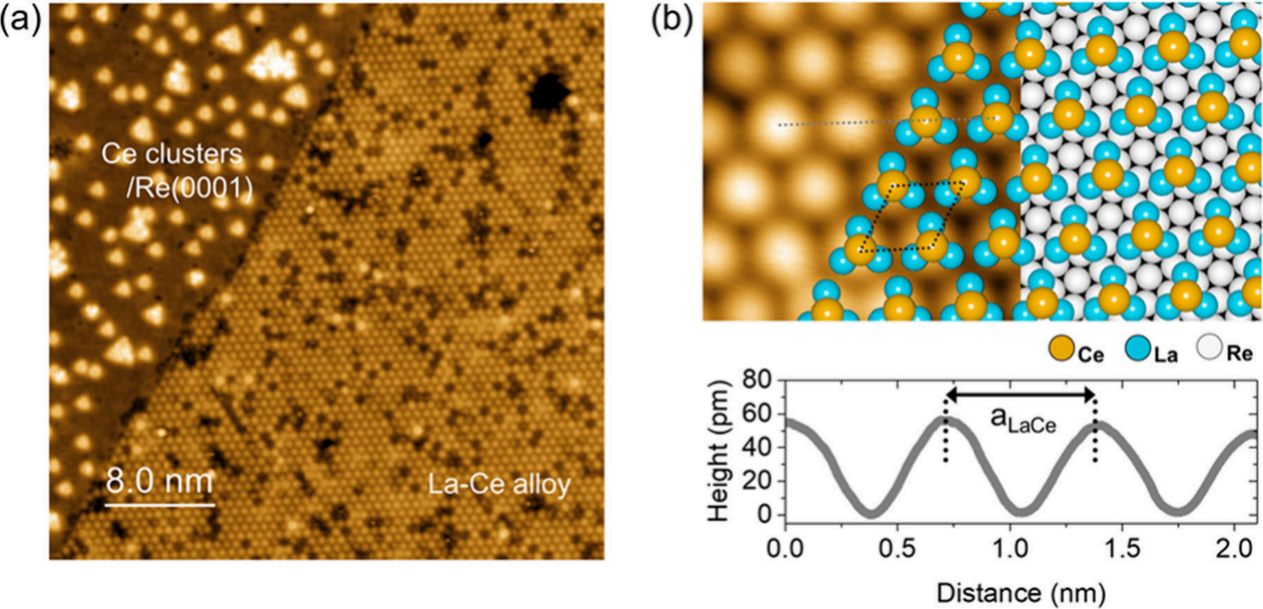
(a) Constant-current STM topography image (40 × 40 nm^2^) showing the formation of an ultrathin La–Ce alloy layer on a Re(0001) substrate (right part). Without La, the Ce forms clusters on the Re(0001) surface (left part). (b) (Top) A zoomed-in STM image revealing the atomic-scale structure of the 2D La–Ce alloy with an atomic ball model superimposed. The unit cell of the La–Ce lattice structure is highlighted by a dotted rhombus. (Bottom) Surface profile along the dotted gray line in (b). The lattice constant (*a*_*LaCe*_) is 0.71 nm which corresponds to √7a_Re_, where *a*_*Re*_= 0.27 nm is the lattice constant of the Re(0001) substrate. Tunneling current: *I*_T_ = 1.0 nA; applied sample bias voltage: *V*_S_ = +40 mV.

To elucidate the electronic structure of the 2D La–Ce alloy in the normal-conducting state of the Re(0001) substrate, we obtained differential tunneling conductance (*dI/dV*) spectra at a temperature T above the superconducting transition temperature of the Re substrate (*T*_*c*,*Re*_ ∼ 1.6 K). Figure 2(a) shows tunneling spectra obtained at T = 1.7 K on a defect-free region of the 2D La–Ce alloy layer (red) as well as on a bare Re(0001) surface (gray). Within the energy range of ±0.04 eV, the La–Ce layer reveals an anomalous spectral feature around the Fermi energy (E_F_), which is absent on the Re(0001) surface. In Figure 2(b), the spatially averaged *dI/dV* spectrum within a smaller energy window shows a broad peak with a dip at E = −0.24 meV resulting in an asymmetric double-peak structure. This asymmetric double-peak feature in the local density of states (LDOS) around the Fermi energy *E*_*F*_ is a characteristic signature of a Kondo lattice.^30−32^ Indeed, we find that the Kondo lattice model provides a good fit to our experimental result, reproducing the asymmetric double peak spectral shape [see Supplementary Note 2] and allowing us to extract a Kondo temperature of 95.2 K. The peak-to-peak distance is determined to be 21.9 meV, which represents the magnitude of the hybridization gap (Δ_hyb_) in the LDOS for a Kondo lattice system.

**Figure 2:**
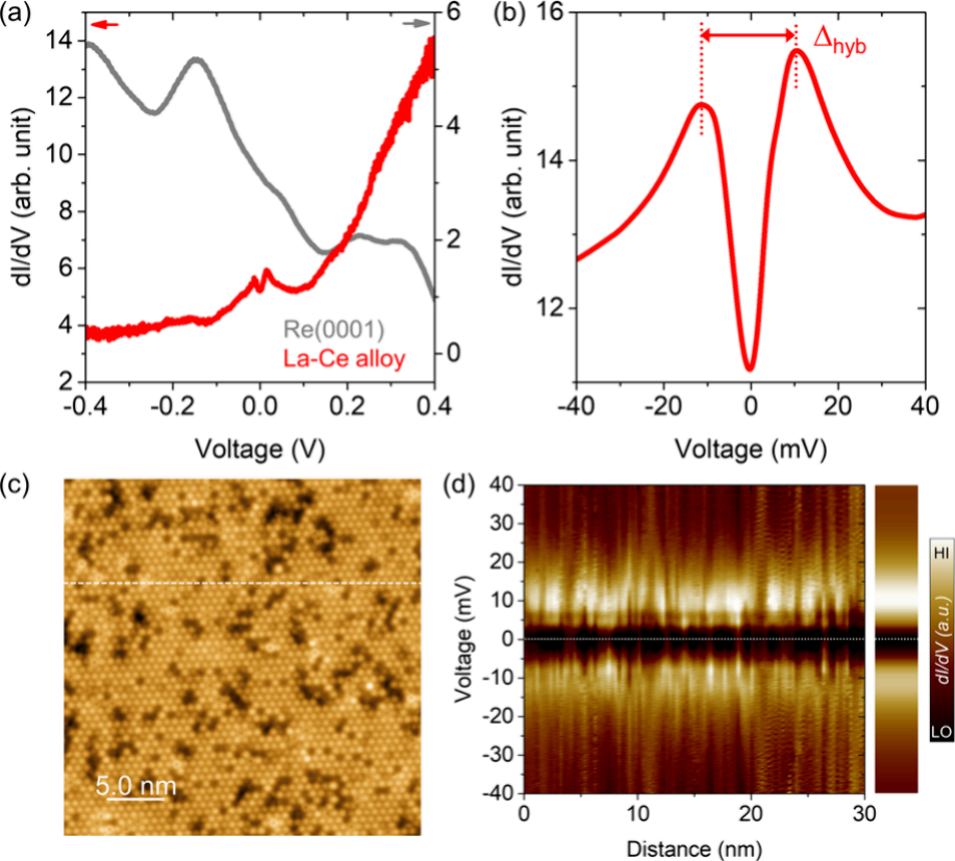
(a) Representative local tunneling spectra taken on the ultrathin La–Ce alloy layer (red, 1.0 nA/-400 mV) and on the uncovered, clean Re(0001) surface (gray, 1.0 nA/+600 mV). (b) Local tunneling spectrum averaged over an extended area of the 2D La–Ce layer revealing an asymmetric double-peak feature around the Fermi level. The peak-to-peak distance corresponding to the hybridization gap Δ_hyb_ is 21.9 meV, and the dip-center is located at energy *E* = −0.24 meV. (c) Constant-current STM topography image (1.0 nA/50 mV, 30 × 30 nm^2^) of the La–Ce alloy layer corresponding to the location where the tunneling spectroscopic data has been obtained. (d) Tunneling spectroscopic map obtained on the 2D La–Ce layer along the dashed line in (c) revealing a robust hybridization gap Δ_hyb_ over the entire area. For comparison, the spectrum in (b) is plotted in the same color-scale (right side). All STM and STS data shown is this figure were obtained at a sample temperature *T* = 1.7 K in the normal conducting state of the Re(0001) substrate.

In order to explore the spatial distribution of the spectral feature of the 2D La–Ce alloy layer, we show a spectroscopic line profile across the La–Ce layer including local defects (see Figure 2(c)), as shown in Figure 2(d). A nearly uniform hybridization gap around E_F_ is revealed with small spatial fluctuations caused by the presence of the local defect sites. For comparison, the averaged tunneling spectrum of Figure 2(b) is plotted on the right-hand side of Figure 2(d). The nearly uniform spatial distribution of the hybridization gap shows that a coherent Kondo lattice state delocalized over the 2D La–Ce alloy film has formed.

Next, we investigate the evolution of the electronic structure of the La–Ce layer as superconductivity of the Re(0001) substrate emerges below the critical temperature *T*_*c*,*Re*_. In Figure 3(a) we present a comparison of the local tunneling spectra of the 2D La–Ce alloy obtained above (gray) and below *T*_*c*,*Re*_ (blue). The spectrum below *T*_*c*,*Re*_ reveals the formation of a proximity-induced superconducting gap around E_F_ and the emergence of associated coherence peaks, while the hybridization gap due to the Kondo lattice state remains almost unaffected. The latter is expected since the hybridization gap is significantly larger than the superconducting order parameter Δ_Re_ in the pure Re compound, such that the proximity-induced superconducting gap is only a small perturbation to the Kondo screened electronic structure.

**Figure 3:**
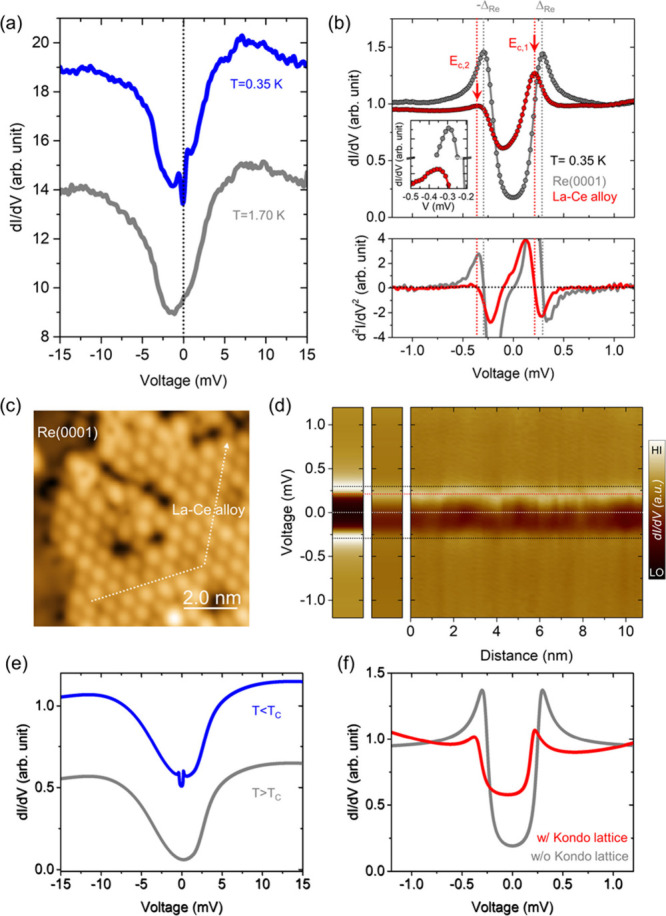
(a) Local tunneling spectrum (blue) obtained on the 2D La–Ce alloy layer at a temperature of 0.35 K, i.e., below *T*_*c*,*Re*_. For comparison, the tunneling spectrum for the same energy range is shown at a temperature of 1.7 K, i.e. above *T*_*c*,*Re*_. The superconductivity-induced spectral feature is visible around *E*_F_ when *T* is below *T*_*c*,*Re*_. (b) Spatially averaged, low-energy tunneling spectra measured on the La–Ce alloy layer (red) and on the uncovered Re(0001) surface near a La–Ce layer (gray). A pronounced peak inside the superconducting gap of Re appears at *E*_c,1_ = +0.22 meV. (Inset) A zoom-in focusing on the small energy range and corresponding spectral shape around the coherence peak at negative bias voltage. (Bottom) Numerical differentiation of the spectra clearly indicating the energy positions of the peaks in the *dI/dV* spectra at *d*^2^*I/dV*^2^ = 0 (red- and gray-dotted lines for the spectra on the La–Ce alloy and Re(0001) surface, respectively). (c) Constant-current STM topographic image of a La–Ce alloy region (right part) next to the bare Re(0001) surface (left part). (d) Tunneling spectroscopic map obtained on the La–Ce alloy region along the dotted line in (c) (right). For direct comparison, the spatially averaged tunneling spectrum for the La–Ce alloy layer (middle) and the bare superconducting Re(0001) substrate (left) are plotted using the same color-scale. Black-, white- and red-dotted lines for ± Δ_Re_, E_F_ and E_c,1_, respectively (e) Theoretical LDOS above (gray) and below (blue) *T*_*c*_. (f) Low-energy LDOS below *T*_*c*_ in the presence (red) and absence (gray) of the Kondo lattice. For details of the theoretical model, see Supplementary Note 3. For details about the experimental procedure, see Supplementary Note 5. Tunneling parameters: (a) *I*_T_ = 1.0 nA, *V*_S_ = 15 mV, and *V*_ac_ = 0.30 mV_rms_; (b, c, and d) *I*_T_ = 0.8 nA, *V*_S_ = +1.2 mV, and *V*_ac_ = 0.03 mV_rms_.

In Figure 3(b), we present a comparison of the averaged low-energy tunneling spectra for the pure Re(0001) surface, and for the ordered La–Ce alloy layer. It is remarkable that while the positions of the coherence peaks are as expected symmetric in energy at ± Δ_*Re*_ on the Re surface, the peaks on the La–Ce alloy layer are shifted downward in energy and are located at asymmetric energies, *E*_*c*,1_ ≠ – *E*_*c*,2_ with *E*_*c*,1_ = +0.21 meV and *E*_*c*,2_ = −0.37 meV [inset of Figure 3(b)]. While this asymmetry is quite unexpected since it apparently breaks the particle - hole symmetry of the superconducting state, the fact that these peaks disappear above *T*_*c*_ implies that they are directly related to the onset of superconductivity, and thus are indeed the superconducting coherence peaks. Further evidence supporting this conclusion comes from spatially resolved tunneling spectra across the La–Ce layer on Re(0001). The spectroscopic line profile shown on the right-hand side of Figure 3(d) along the dotted line in the STM image of Figure 3(c) reveals a pronounced peak at *E*_*c*,1_ over the entire line, indicating that it is not related to some local defect state (in which case the particle- and hole-like components should exhibit spatial out-of-phase oscillations,^45,46^ which are not observed), but that this peak represents a coherent feature of the Kondo lattice in which superconductivity is proximity-induced. For comparison, an averaged spectrum is shown in the middle of Figure 3(d), which is clearly distinct from the one measured above an uncovered Re(0001) area (left part of Figure 3(d)).

A first clue as to the origin of the asymmetry in the energy positions of the coherence peaks comes from the observation that both peaks are uniformly shifted to lower energy by Δ*E* = 80 μeV with respect to the energetically symmetric coherence peaks at ± Δ_*Re*_ = 0.29 meV in the spectrum of bare superconducting Re(0001) (gray), i.e., *E*_*c*,1,2_*=* ± Δ_*Re*_*–*Δ*E*. Such a uniform shift can arise from the presence of residual (unscreened) magnetic moments, and the formation of a YSR band,^38,40^ as discussed in detail below.

To understand the experimentally observed uniform shift of the Re coherence peaks to lower energies in the La–Ce alloy, we note that the observed Kondo resonance in the larger energy window most likely arises from the coupling of the localized *f*-orbital-derived magnetic moments of Ce with the itinerant conduction (*c*-) electrons, and the concomitant coherent Kondo screening of the Ce moments. While it is presently unclear whether the Ce moments are fully screened above *T*_*c*_, the opening of a superconducting gap below *T*_*c*_, and the concomitant gapping of the conduction band’s low-energy degrees of freedom, is expected to increase the magnitude of the (partially) unscreened Ce moments. To understand the effect of these unscreened moments on the system’s electronic structure, we consider a large-N theory for the Kondo lattice,^6,19,47−50^ using a generic two-band model, with proximity-induced superconductivity, and a residual unscreened effective moment of magnitude *S* that interacts with the heavy *f*-electron (conduction electron) states via a Heisenberg exchange *I* (*J*) [see Supplementary Note 3]. The largest effect of this interaction occurs for states near the Fermi energy, which in the generic mean-field approximation of the Kondo lattice model, are predominantly of *f*-electron character [see Supplementary Figure S3(a) and the discussion on the hybridization of dispersive bands in Supplementary Note 3]. As a result, the partially unscreened magnetic moment leads to a uniform energy shift of Δ*E*= ± *IS* in the electronic structure of the two spin species of the *f*-electrons. This shift can be interpreted as the formation of YSR bands of predominant *f*-electron character on the background of the partially Kondo screened and superconducting La–Ce/Re hybrid system [see Supplementary Figure S3(b) and the discussion on hybridized bands in the presence of superconductivity in Supplementary Note 3]. The fact that the experimental results reveal only a uniform downward shift of the electronic structure to negative energies can be explained by assuming that the STM tip itself is spin-polarized, possibly by picking up Ce atoms, thus leading to a preferential tunneling of electrons of only one spin-polarization [see Supplementary Figure S4(f) and the discussion on the role of spin-polarized tips in Supplementary Note 3]. By reversing the spin-polarization of the tip, the asymmetry of the peak intensities is reversed and the corresponding peak positions are shifted upward [see Supplementary Note 4 for details].

The resulting theoretical *dI*/*dV* in the normal state above *T*_*c*_ shown in Figure 3(e) reproduces all salient features of the experimental *dI*/*dV* in Figure 3(a): a hybridization gap of about 20 meV, a slightly asymmetric Kondo resonance, and a minimum in the LDOS around zero energy. Below *T*_*c*_, a superconducting gap opens on the background of the Kondo resonance. In Figure 3(f), we compare the theoretically obtained low-energy *dI*/*dV* in the absence of a Kondo lattice (corresponding to the pure Re surface) and in the presence of a Kondo lattice with a partially unscreened magnetic moment (corresponding to the LaCe-alloy) assuming a spin-polarized STM tip. The combination of a spin-polarized tip together with the presence of the unscreened moment results in coherence peaks that are located at asymmetric energies *E*_*c*,1_ ≠ *–E*_*c*,2_, in agreement with the experimental results shown in Figure 3(b). A comparison of the *dI*/*dV* obtained in the presence or absence of unscreened moments, with an unpolarized or spin-polarized tip (see Supplementary Figure S4 in Supplementary Note 3) shows that this energy asymmetry of the coherence peaks is a unique and direct signature for the presence of unscreened magnetic moments, and the predominant tunneling into one of the spin channels due to a spin-polarized tip state, as discussed above (see Supplementary Note 4 for the experimental verification of spin-polarized tips).

Finally, we investigate the relationship between the energy of the coherence peaks at *E*_*c*,1,2_ and the magnitude of the hybridization gap (Δ_hyb_), and present in Figure 4(a) a correlation map of (Δ_hyb_, *E*_*c*,1_) extracted from local tunneling spectra above the ordered 2D La–Ce alloy (red) and above edge- or defect-sites (gray). This map reveals a positive correlation between Δ_hyb_ and *E*_*c*,1_ for the ordered 2D La–Ce alloy as indicated by the red ellipse: *E*_*c*,1_ moves to higher energies and thus closer to the superconducting gap edge Δ*Re* of the pure Re(0001) surface with increasing Δ_hyb_. On the other hand, no correlation with Δ_hyb_ is found when considering *E*_*c*,1_ near edge- and defect-sites of the La–Ce layer, where the observed sizable and random variation in *E*_*c*,1_ likely arises from an inhomogeneous distribution of unscreened magnetic moments.

**Figure 4:**
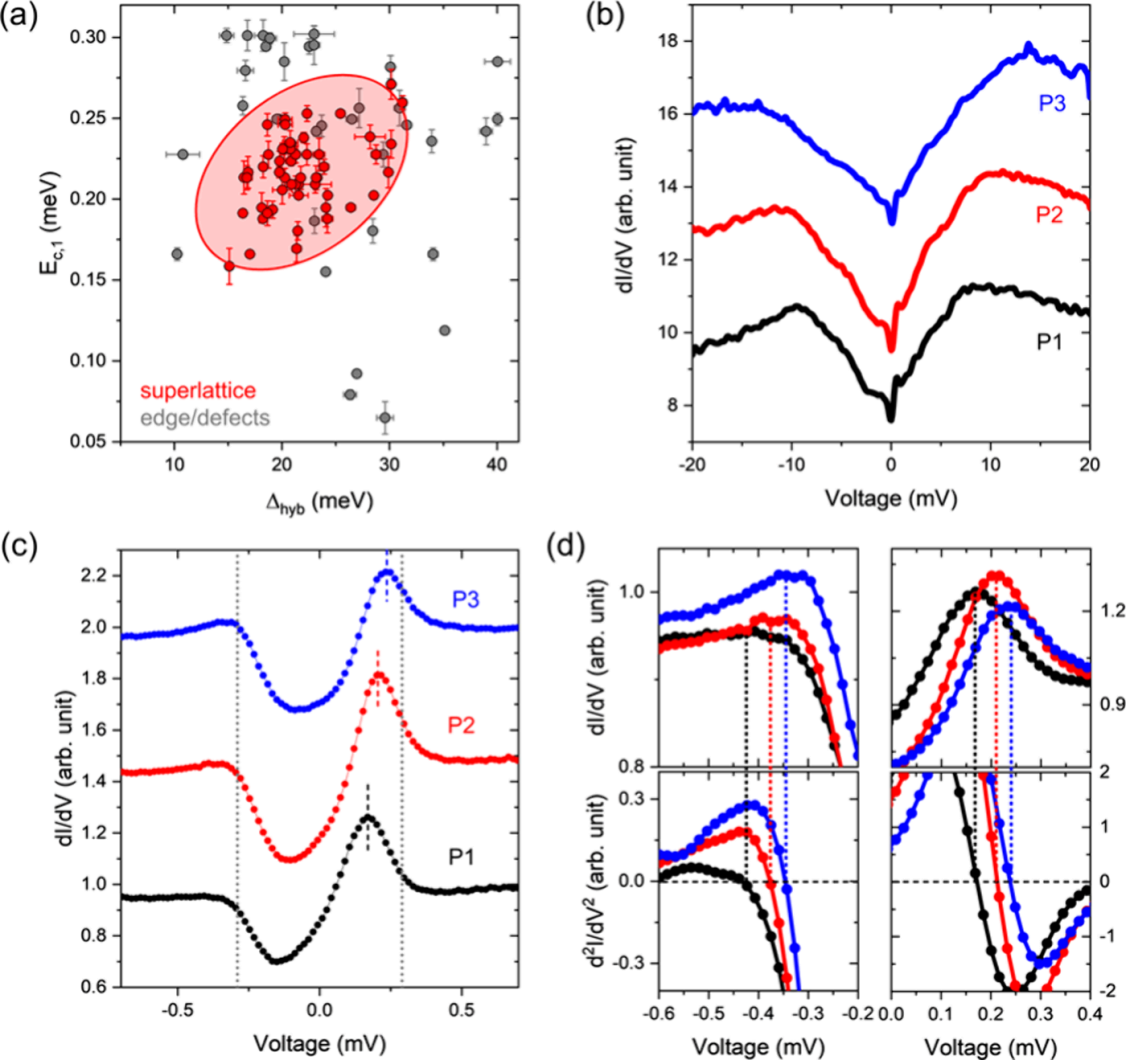
(a) Correlation map showing the shifted coherence peak *E*_c,1_ as a function of the hybridization gap Δ_hyb_ extracted from local tunneling spectra obtained on the ordered La–Ce alloy layer (red) and edge/defect-sites of the La–Ce layer (gray). A positive correlation is apparent as marked with a tilted 95%-confidence ellipse (shaded in red) for points on the ordered La–Ce layer. (b) and (c) Representative local tunneling spectra obtained at three different spatial locations (P1–P3) with a larger energy window to extract Δ_hyb_ (b), and a smaller one to extract E_c,1_ (c). Colored dashed lines indicate the values for E_c,1_, whereas the gap edges are depicted with gray dotted lines at *E* = 0.29 meV. (d) The same plots as depicted in (c) with a numerical differentiation of the spectra (bottom) to determine the energy values of *E*_c,1_ and *E*_c,2_. The dotted lines are indicating the peak positions for each measured *dI/dV* spectrum. All tunneling spectra were obtained at *T* = 0.35 K. Tunneling parameters: (b) *I*_T_ = 1.0 nA, *V*_S_ = 20 mV, and *V*_ac_ = 0.3 mV_rms_; (c) *I*_T_ = 0.8 nA, *V*_S_ = +1.2 mV, and *V*_ac_ = 0.03 mV_rms_.

To further demonstrate the positive correlation for the ordered La–Ce alloy based on primary STS data, we present two sets of tunneling spectra obtained at three different sites (P1 - P3) of the ordered 2D La–Ce alloy in Figure 4(b) and 4(c), respectively. As Δ_hyb_ increases between P1 to P3 (Figure 4(b)), *E*_*c*,1_ gradually shifts toward higher energies and to the gap edge of the pure Re(0001) surface [Figure 4(c)]. Even more revealing is the fact that *E*_*c*,2_ shifts in unison with *E*_*c*,1_ [see Figure 4(d)], such that for all sites (P1 - P3) *E*_*c*,1_–*E*_*c*,2_ = 2Δ_*Re*_. This correlation reflects that with increasing strength of the Kondo screening (resulting in a larger Δ_hyb_), the partially unscreened moment is reduced, diminishing the shift Δ*E* = *IS* of *E*_*c*,1,2_ with respect to the coherence peaks of the pure Re located at ± Δ_*Re*_.

While the competition between Kondo screening and superconductivity is still far from understood for a Kondo lattice system, such as the ones discussed here, it has been extensively discussed for a single magnetic impurity. In this case, the competition is characterized by the Kondo temperature *T*_*K*_ (corresponding to the width of the Kondo resonance which is the precursor of the Kondo lattice hybridization gap), the superconducting gap Δ, and the binding energy E_YSR_ of the YSR state induced by the partially unscreened magnetic moment. According to Matsuura’s model based on the local Fermi-liquid approach for the strong Kondo regime (Δ ≪ *k*_*B*_*T*_*K*_)^51^ which is realized in our experiment, the energy of the YSR bound states was found to be
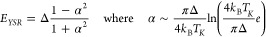


Thus, with increasing *T*_*K*_ (resulting in an increased width of the Kondo resonance), α decreases and *E*_*YSR*_ moves closer to the edge of the superconducting gap due to a smaller unscreened moment. This is qualitatively similar to our experimental finding that with increasing Δ_hyb_, *E*_*c*,1,2_ moves closer to the coherence peaks of the pure Re(0001) surface at ± Δ_*Re*_, thus providing further support for our interpretation.

In summary, we have explored a novel type of 2D La–Ce alloy epitaxially grown on a Re(0001) substrate. Our detailed STS investigations have revealed clear spectroscopic signatures of a Kondo lattice state above and below the critical temperature *T*_*c*,*Re*_ of Re, as well as the emergence of a proximity-induced superconducting gap below *T*_*c*,*Re*_ in the La–Ce alloy. We show that our observation of coherence peaks which are located at asymmetric energies can be consistently explained as arising from the interplay between the presence of partially unscreened magnetic moments and a spin-polarized tip. Additionally, we identify a positive correlation between the hybridization gap Δ_hyb_ and the energy position of the coherence peaks, *E*_*c*,1_,_2_ with *E*_*c*,1,2_ shifting closer to the coherence peaks of the pure Re at ± Δ_*Re*_ with increasing Δ_hyb_, indicating that the residual magnetic moments are reduced with increasing strength of the Kondo screening. For the future, it could be interesting to perform STM/STS investigations on other material systems of low-dimensional Kondo lattices proximitized to superconducting substrates, where the energy scale of the Kondo hybridization is comparable to or even smaller than that of the superconducting pairing, which is the opposite limit compared to the present study. Our results provide novel insight into the behavior of low-dimensional Kondo lattice–superconductor hybrid systems, which can become a versatile platform for studying microscopic aspects of exotic quantum states in artificially designed superconducting *f*-electron based materials.

## Methods

### Sample and STM Tip Preparation

The Re(0001) single crystal used as a superconducting substrate in this work was prepared by repeated cycles of O_2_ annealing at 1400 K followed by flashing at 1800 K to obtain an atomically flat Re(0001) surface.^52^ Lanthanum and cerium were deposited separately *in situ* under ultrahigh vacuum conditions by electron beam evaporation of pure La pieces (99.9+%, MaTeck, Germany) and a Ce rod (99.9+%, MaTeck, Germany) from molybdenum crucibles. The deposition rates of both materials were calibrated separately by depositing them onto a clean Re(0001) surface prior to the preparation of the ultrathin La–Ce alloy layer. The 2D La–Ce alloy was prepared by a two-step *in situ* process. Initially, a monolayer of La was deposited onto the Re(0001) substrate followed by annealing at 950 K for 5 min, resulting in the formation of a La wetting layer on the Re(0001) surface. Subsequently, a submonolayer coverage of Ce was deposited onto the La wetting layer followed by annealing at 950 K for 15 min. Finally, the samples were transferred into the cryostat without breaking vacuum. Commercially available Pt–Ir tips were used as STM probes being sharpened and cleaned by *in situ* tip treatments. Such Pt–Ir tips do not exhibit a magnetization. However, the tip apex can become magnetic by picking up Ce atoms from the Ce-covered Re(0001) surface by the STM tip.

### STM/STS Measurements

All STM/STS experiments were performed in a ^3^He-cooled low-temperature STM system (USM-1300S, Unisoku, Japan) operating at *T* = 0.35 K up to 1.70 K under ultrahigh vacuum conditions. Tunneling spectra were obtained by measuring the differential tunneling conductance (*dI/dV*) using a standard lock-in technique under opened feedback loop with a frequency of 1128 Hz and modulation voltages of 0.03 mV_rms_ and 0.30 mV_rms_ in order to resolve the spectral features related with superconductivity and Kondo hybridization, respectively. The bias voltage was applied to the sample and the tunneling current was measured through the tip using a commercially available controller (Nanonis, SPECS).

## Supporting Information Available

Atomic-scale STM image of the ultrathin La–Ce alloy film in the vicinity of a Ce vacancy island with corresponding atomic structure model, the extraction of the Kondo temperature based on the Kondo lattice model, the theoretical model, information about tunneling spectroscopic measurements, and the experimental demonstration of spin polarization effects (PDF)
