# Supplementary material for: Proximity-Induced Superconductivity in a 2D Kondo Lattice of an f-Electron-Based Surface Alloy
Source: Nano Lett. 2024 Oct 25;24(44):14139–45. doi: 10.1021/acs.nanolett.4c04796 (PMC11544701; doi:10.1021/acs.nanolett.4c04796)
Supplement: Supplementary file 1 — nl4c04796_si_001.pdf [file nl4c04796_si_001.pdf]

Supporting Information for

Proximity-induced superconductivity in a 2D Kondo lattice of an *f*-electron-based surface alloy

Howon Kim[1,*,†], *Dirk K. Morr*[2] *and Roland Wiesendanger*[1,*]

[1]Department of Physics, University of Hamburg, D-20355 Hamburg, Germany

[2]Department of Physics, University of Illinois at Chicago, Chicago, IL, 60607, USA

Corresponding Authors(*): hkim@physnet.uni-hamburg.de and wiesendanger@physnet.uni-hamburg.de

Supplementary Note 1. Atomic-scale STM image of the ultrathin La-Ce alloy film in the vicinity of a Ce vacancy island with corresponding atomic structure model

To understand the atomic structure of the 2D La-Ce alloy, we have focused on vacancy defects in the La-Ce layer, which perturb the hexagonal lattice structure. We assume that these vacancy defects occur in the La-Ce layer due to an inhomogeneous concentration distribution of La and Ce atoms during the Ce deposition process. Additionally, the bonding strength between La and Ce could be relatively weak resulting in the local evaporation of Ce atoms into the vacuum during the annealing process. In Figure S1(a), a vacancy island is visible in the middle of the La-Ce layer. In the vicinity of the vacancy island, we observe an extended defect in the La-Ce layer with a slightly darker contrast. Figure S1(b) shows a zoomed-in STM image (a dotted square in Fig. S1(a)) focusing on the extended defect. A trimer-like structure is visible at the vacancy sites of the lattice formed by the bright protrusions. Based on the trimer-like structure as revealed by STM and the consideration of the deposited amount of La and Ce, we arrive at an atomic structure model as shown in Fig. 1(b), which basically corresponds to an ordered hexagonal array of La_3Ce complexes. This model is consistent with the observed asymmetric double-peak resonance in the differential tunneling conductance spectra measured on the 2D La-Ce layer, reflecting that a large portion of electrons from the STM tip tunnel into the localized *f*-orbital localized at the Ce atoms being next to the tip apex.

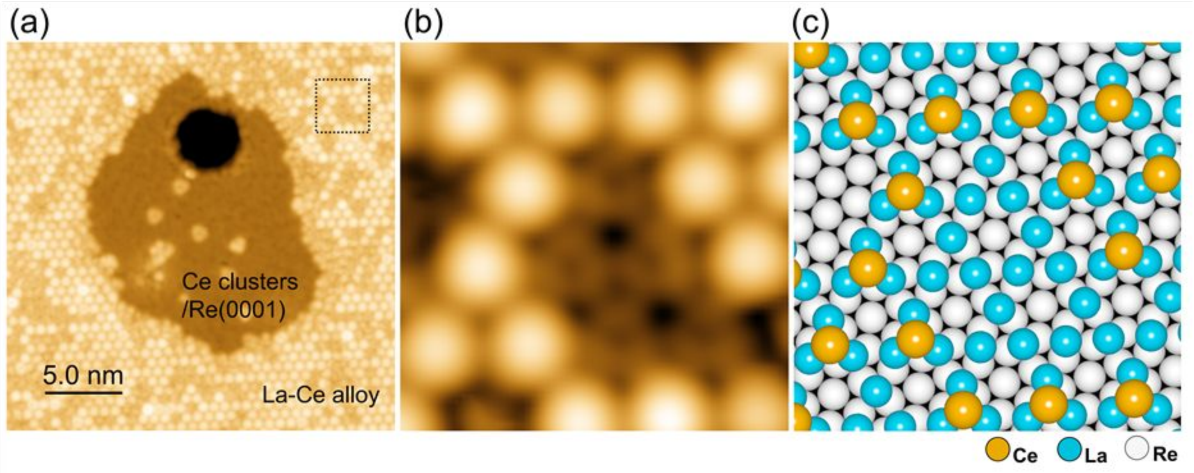

Supplementary Figure S1. (a) Constant-current STM topography image (25 × 25 nm^2) showing a vacancy island of the 2D La-Ce alloy at T = 1.7 K (1.0 nA/1.5 mV). The vacancy island shows the same characteristics as the terrace of the bare Re(0001) surface as shown in Fig. 1. (b) A zoomed-in STM image of the marked area (dotted square in (a)) revealing trimer-like structures at Ce-vacancy sites (25 × 25 nm^2). Holes shown as dark depressions are visible besides the trimer-like structures. (c) Atomic structure model based on the STM image in (b). Note that the trimer-like structures are formed by La atoms being in direct contact with the Re(0001) substrate. Each La trimer binds one Ce atom, thereby forming a La_3Ce complex. These La_3Ce complexes form an ordered hexagonal lattice on Re(0001).

Supplementary Note 2. Extraction of the Kondo temperature for La-Ce alloy layer

Fig. S2 shows the theoretical fit to the measured tunneling spectrum based on the extended Fano theory for a Kondo lattice. By adopting the extended Fano analysis based on the co-tunneling process to a Kondo lattice system, the differential tunneling conductance can be expressed by [1]:

$$\frac{dI}{dV_{KL}} = \left(1 + \frac{q_{KL}W}{eV-\lambda}\right)^2 \ln\left[\frac{eV + D_1 - \frac{v^2}{eV-\lambda}}{eV - D_2 - \frac{v^2}{eV-\lambda}}\right] + \frac{2D/t_c^2}{eV-\lambda} \quad \text{, where} \quad q_{KL} = t_f v / t_c W$$

Here, λ is the renormalized resonance position, $D_1$and $D_2$are the lower and upper conduction band edges, W is the width of the resonance, v is the hybridization amplitude, and q_{KL} is the co-tunneling parameter described by the tunneling probability to the *f*-level, t_f, and the tunneling probability to the conduction band, t_c.

The extracted indirect hybridization gap, $\Delta_h = \frac{2v^2}{(D_1+D_2)/2}$ is 8.2 meV, i.e. 95.2 K with the parameters, t_f= 1.4, t_c=3.28, D_1=120, D_2=97, v =0.681, W =0.08. The good agreement between the experimental dI/dV spectrum and the theoretical fit based on the Kondo lattice model provides strong support for our description of the La-Ce alloy as a coherent Kondo lattice above T_c. According to mean-field theory, the energy scale of the indirect hybridization gap in the local density of states is approximately the same as the energy scale of the Kondo temperature for a single Kondo impurity, $k_B\Delta_h \sim T_K$.

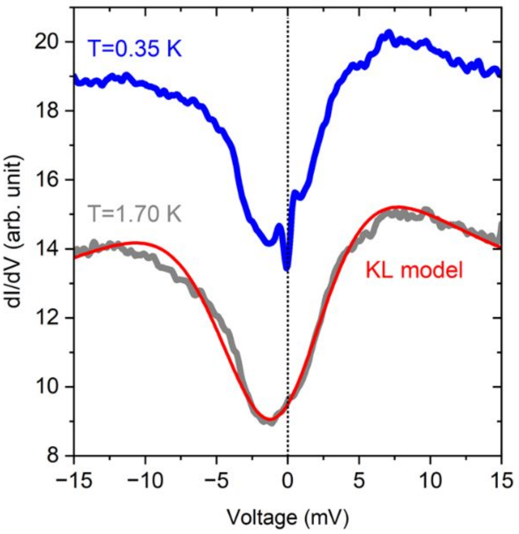

Supplementary Figure S2. Tunneling spectra of Fig. 3(a) in the main text with the simulated curve based on the Kondo lattice (KL) model (red).

Supplementary Note 3. Theoretical model

To compute the electronic and spectroscopic structure of the Kondo screened magnetic layer above T_c, our starting point can either be a Hamiltonian in the band basis, or one in the orbital basis. Since the physical properties near the Fermi energy are dominated by the heavy magnetic states, we show below that both starting points lead to an essentially identical low-energy structure. For simplicity, we therefore start with a two-dimensional (2D) Hamiltonian that yields a description of the low-energy physics in terms of a band basis given by [2]:

$$H = \sum_{\mathbf{k},\sigma} \varepsilon_{\mathbf{k}} c^\dagger_{\mathbf{k}\sigma} c_{\mathbf{k}\sigma} + \sum_{\mathbf{k},\sigma} \chi_{\mathbf{k}} f^\dagger_{\mathbf{k}\sigma} f_{\mathbf{k}\sigma} + V \sum_{\mathbf{k},\sigma} \left(f^\dagger_{\mathbf{k}\sigma} c_{\mathbf{k}\sigma} + c^\dagger_{\mathbf{k}\sigma} f_{\mathbf{k}\sigma} \right) \quad (1)$$

where

$$\varepsilon_{\mathbf{k}} = \; -2\, t_c\left[cos(k_x) + cos(k_y)\right] - \mu_c \tag{2}$$

$$\chi_k = -2t_f\left[cos(k_x) + cos(k_y)\right] - \mu_f \tag{3}$$

are the dispersions of the light conduction electrons ($\varepsilon_{\mathbf{k}}$) and heavy magnetic states (χ_k), and V is the hybridization between them. Here, $c^{\dagger}_{\mathbf{k}\sigma}$ ($f^{\dagger}_{\mathbf{k}\sigma}$) creates an electron with momentum $\mathbf{k}$ and spin σ in the light (heavy magnetic) bands. This Hamiltonian is obtained from the $U \rightarrow \infty$ limit of the Anderson Kondo lattice Hamiltonian [3–5] and describes only the surface of the system. Note that the dispersion of the heavy magnetic states (also referred to as the f-electron states) in general arises from a Heisenberg exchange coupling I between neighboring magnetic atoms [2]. Diagonalizing the above Hamiltonian yields

$$H = \sum_{\mathbf{k},\sigma} \left(E^{\alpha}_{\mathbf{k}} \alpha^{\dagger}_{\mathbf{k}\sigma} \alpha_{\mathbf{k}\sigma} + E^{\beta}_{\mathbf{k}} \beta^{\dagger}_{\mathbf{k}\sigma} \beta_{\mathbf{k}\sigma} \right) \tag{4}$$

where

$$E^{\alpha,\beta}_{\mathbf{k}} = \frac{\varepsilon_{\mathbf{k}} + \chi_{\mathbf{k}}}{2} \pm \sqrt{\left(\frac{\varepsilon_{\mathbf{k}} - \chi_{\mathbf{k}}}{2}\right)^2 + V^2} \tag{5}$$

For the theoretical results shown in the main text, we used the following parameters:

$t_c = 100\; meV, \mu = -3.618t_c, t_f = 0.025t_c, \mu_f = 0.07t_c, V = 0.3t_c,$ yielding the dispersion shown in Fig. S3(a) in the normal state. We assume that superconductivity is proximity induced in the La-Ce layer on the background of these two bands, which are formed at temperatures much higher than T_c. In general, only one of the two bands, $E^{\beta}_{\mathbf{k}}$, crosses the Fermi surface [see Fig. S3(a)], and we thus assume that only this band becomes superconducting via proximity coupling to the bulk Re system below T_c,

As we discuss in the main text, and show below, the experimental dI/dV results reveal strong evidence for the presence of a partially unscreened f-electron moment S. Thus, since the electronic states near the Fermi surface are predominantly of heavy f-electron character, as follows from Fig. S3(a), the main effect of the unscreened f-electron magnetic moment arises from its coupling to heavy f-electron states via the Heisenberg exchange coupling I. Thus, only the bands $E^{\beta}_{\mathbf{k}}$ crossing the Fermi surface will be subject to an effective Zeeman splitting by $\pm IS$. The Hamiltonian below T_c then takes the form

$$H = \sum_{\mathbf{k},\sigma} E^{\alpha}_{\mathbf{k}} \alpha^{\dagger}_{\mathbf{k}\sigma}\alpha_{\mathbf{k}\sigma} + \sum_{\mathbf{k},\sigma} \left(E^{\beta}_{\mathbf{k}} - IS\mathrm{sgn}\sigma\right)\beta^{\dagger}_{\mathbf{k}\sigma}\beta_{\mathbf{k}\sigma} + \sum_{\mathbf{k}} \Delta\left(\beta^{\dagger}_{\mathbf{k}\uparrow}\beta^{\dagger}_{\text{-}\mathbf{k}\downarrow} + \beta_{\text{-}\mathbf{k}\downarrow}\beta_{\mathbf{k}\uparrow}\right) \tag{6}$$

where Δ is the proximity induced superconducting order parameter. The resulting dispersion in the superconducting state is shown in Fig. S3(b), and a zoom-in is presented in Fig. S3(c). A comparison of Figs. S3(a) and (c) reveals that the superconducting gap opens as expected in the predominantly f-electron part of the dispersion, since the c-electron dispersion is pushed away from the Fermi energy due to the opening of the hybridization gap. The resulting retarded Greens function for the spin-σ c-electrons is then given by

$$G_{cc}(k,\sigma,\omega) = \frac{u_k^2}{\omega + i\Gamma(\omega) - E^{\alpha}_{\mathbf{k}}} + v_k^2 \frac{\omega \pm IS + i\Gamma(\omega) - E^{\beta}_{\mathbf{k}}}{(\omega \pm IS + i\Gamma(\omega))^2 - \left(\Omega^{\beta}_{\mathbf{k}}\right)^2} \tag{7}$$

with the upper (lower) sign for $\sigma = \uparrow (\sigma = \downarrow)$, and

$$u_k^2, v_k^2 = \frac{1}{2}\left(1 \pm \frac{\frac{\varepsilon_{\mathbf{k}} - \chi_{\mathbf{k}}}{2}}{\sqrt{\left(\frac{\varepsilon_{\mathbf{k}} - \chi_{\mathbf{k}}}{2}\right)^2 + V^2}}\right) \tag{8}$$

and

$$\Omega^{\beta}_{\mathbf{k}} = \sqrt{(E^{\alpha}_{\mathbf{k}})^2 + \Delta^2} \tag{9}$$

To model the experimentally observed broadening in the differential conductance dI/dV, we consider a frequency dependent inverse particle lifetime given by

$$\Gamma(\omega) = \Gamma_0 + \alpha\omega^2 \tag{10}$$

with $\Gamma_0 = 0.03meV$ and $\alpha = 0.238/meV$.

The local density of states (DOS) for the translationally invariant system is then given by

$$N_{cc}(\sigma,\omega) = -\frac{1}{\pi}\mathrm{Im}[G_{cc}(\mathbf{r},\sigma,\omega)] \tag{11}$$

where

$$G_{cc}(\mathbf{r},\sigma,\omega) = \int \frac{d^2k}{(2\pi)^2} G_{cc}(\mathbf{k},\sigma,\omega) \tag{12}$$

To directly compare the theoretical DOS with the experimental dI/dV shown in Fig. 3(b) of the main text on the pure Re and the La-Ce alloy surfaces, we note that the STS experiments

are conducted in closed loop mode, implying that the distance between the tip and the surface is adjusted such that the total current between the tip and the surface for given set point voltage V_s remains constant. As was shown in Ref. [7], this implies that in order to compare the theoretical DOS for the pure superconducting system with that in the presence of Kondo screening, the theoretical $N(\omega)$ needs to be renormalized according to

$$N_{ren}(\omega) = N(\omega)\left[\int_0^{V_s} N(\omega)\right]^{-1} \tag{13}$$

The results for $N_{ren}(\omega)$ are shown in Fig. 3(f) of the main text, where we employed $V_s = 1$ meV.

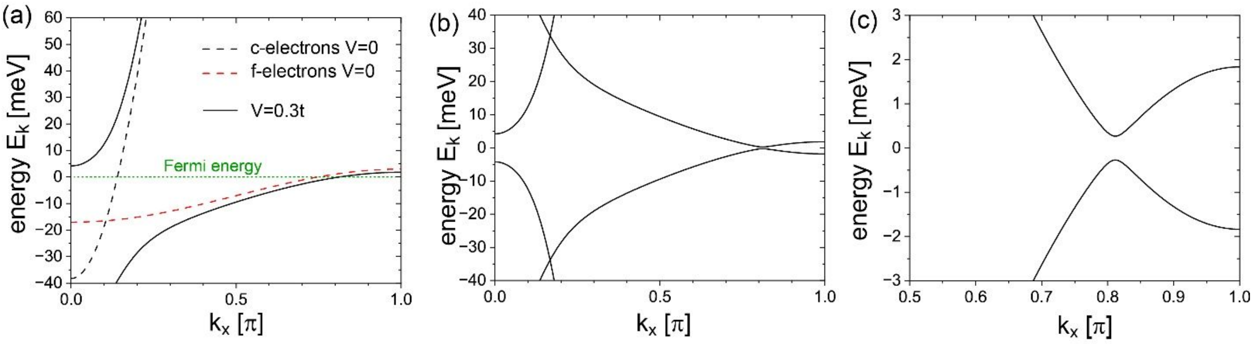

Supplementary Figure S3. (a) Dispersion in the normal state along the diagonal ($k_x = k_y$) in the Brillouin zone, for the unhybridized (V = 0) and hybridized (V = 0.3t) case. (b) Dispersion along the diagonal in the superconducting state with (c) being a zoom-in of (b).

To demonstrate the characteristic features that occur in the LDOS due to a partially unscreened magnetic moment S in conjunction with a spin-polarized STM tip, we present in Fig. S4 a comparison of the LDOS, $N_{cc}(\sigma,\omega)$, and the resulting dI/dV for several different cases. Let us first consider the LDOS for a system with zero magnetization, i.e., $S = 0$, as shown in Fig. S4(a). In this case, the spin-↑ and spin-↓ bands are degenerate, and the LDOS exhibits coherence peaks which are located at symmetric energies, $\pm E_c$. Due to the spin degeneracy, the differential conductance dI/dVmeasured with an unpolarized [see Fig. S4(c)] or spin-polarized STM tip [see Fig. S4(e)] yield identical results (up to an overall factor). Specifically, in the weak-tunneling limit, $dI/dV \sim N_{cc}(\sigma,\omega)$ (assuming that electron tunneling occurs only between the STM tip states and the sample's conduction electron band).

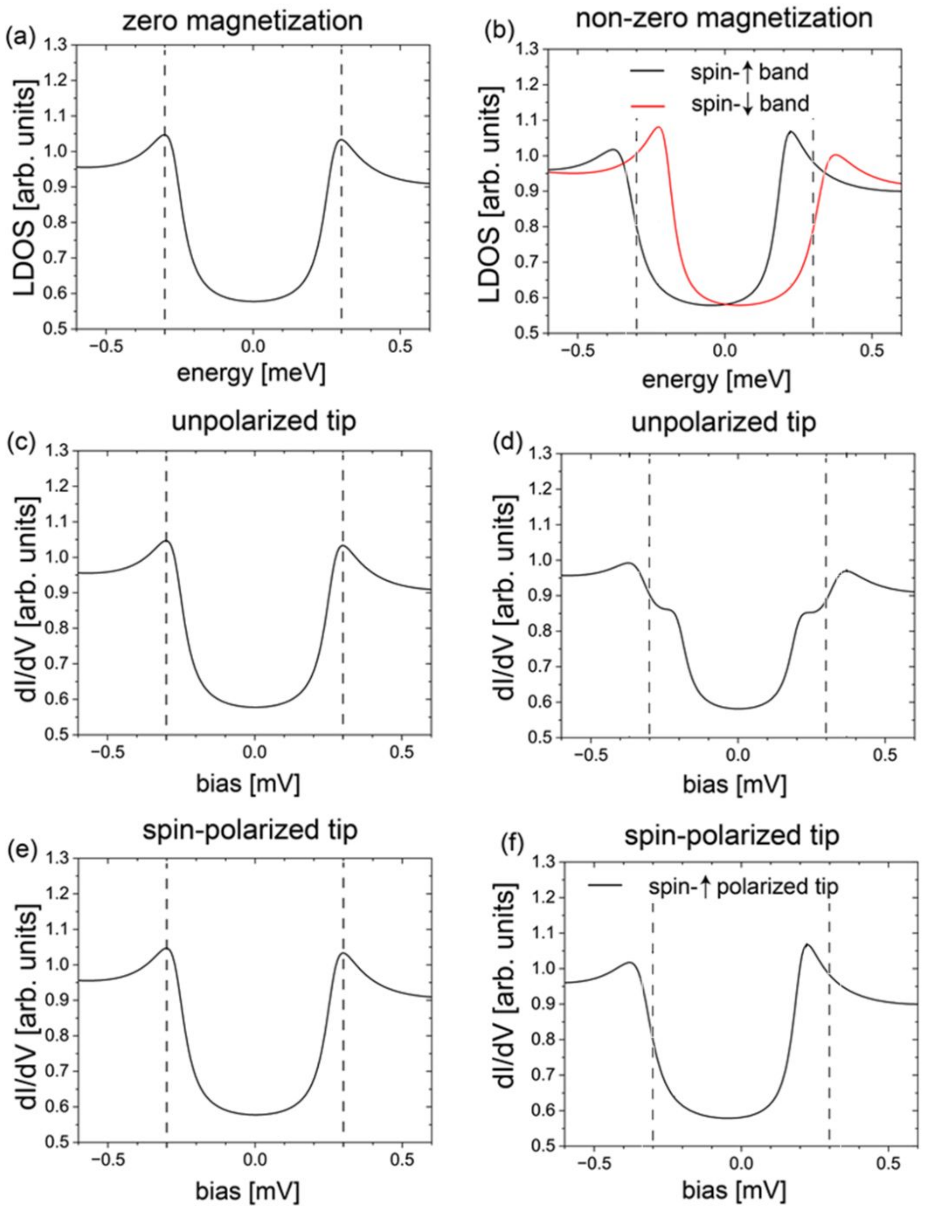

Supplementary Figure S4. A comparison of the LDOS, $N_{cc}(\sigma,\omega)$, and simulated differential conductance, dI/dV, curves. (a) Calculated LDOS $N_{cc}(\sigma,\omega)$ for a system with zero magnetization, i.e., S = 0. (b) Spin-resolved LDOS for S ≠ 0, i.e. for a non-zero magnetization of the Kondo lattice proximitized to the superconducting substrate. (c) and (d) Simulated dI/dV curves assuming an unpolarized STM tip for the two cases S = 0 and S ≠ 0. (e) and (f) Simulated dI/dV curves assuming a spin-polarized tip for the two cases S = 0 and S ≠ 0. Only for the case of a spin-polarized tip and a non-zero magnetization of the Kondo lattice, an asymmetric dI/dV curve (with respect to zero bias) is obtained. Dotted lines in (a)-(f) indicate the peak positions (bias= ±0.3 mV) for the zero magnetization case.

In contrast, in the presence of a non-zero magnetization, $S \neq 0$, the energies of the spin-↑ and spin-↓ bands, and in particular the energy position of the coherence peaks, are shifted by $\pm IS$ [see Eq. (7)], yielding the spin-resolved LDOS shown in Fig. S4(b). Note that this shift can be interpreted as arising from the formation of Yu-Shiba-Rusinov bands. For an unpolarized STM tip, one then obtains $dI/dV \sim N_{cc}(\uparrow,\omega) + N_{cc}(\downarrow,\omega)$, yielding a dI/dV that still exhibits coherence peaks at symmetric energies $E_1 = -E_2$, as shown in Fig. S4(d). However, if the STM tip is fully spin-polarized, tunneling into only one of the two spin bands is possible, leading to a dI/dV curve that is asymmetric in energy, as shown in Fig. S4(f). Specifically, for a spin-↑ polarized tip, the coherence peaks measured in dI/dV are located at asymmetric energies, i.e., $E_1 \neq -E_2$. To the best of our knowledge, the presence of a non-zero magnetic moment in combination with a spin-polarized tip is the only possible explanation for the observation of coherence peaks in the superconducting states that are located at asymmetric energies $E_1 \neq -E_2$. Since only one of the four possible cases shown in Figs. S4(c), (d), (e), and (f) yields the asymmetric energies of the coherence peaks in dI/dV observed experimentally, strong evidence for the presence of not only a non-zero magnetization, but also a spin-polarized STM tip exists.

Finally, we briefly note that an alternative approach, which introduces the proximity induced superconducting order parameter in the orbital basis, yields essentially the same results. This approach starts from the Hamiltonian

$$H = \sum_{\mathbf{k},\sigma} (\varepsilon_{\mathbf{k}} - JS\mathrm{sgn}\sigma) c^{\dagger}_{\mathbf{k}\sigma} c_{\mathbf{k}\sigma} + \sum_{\mathbf{k},\sigma} (\chi_{\mathbf{k}} - IS\mathrm{sgn}\sigma) f^{\dagger}_{\mathbf{k}\sigma} f_{\mathbf{k}\sigma} + V \sum_{\mathbf{k},\sigma} \left(f^{\dagger}_{\mathbf{k}\sigma} c_{\mathbf{k}\sigma} + c^{\dagger}_{\mathbf{k}\sigma} f_{\mathbf{k}\sigma} \right) + \sum_{\mathbf{k}} \left(\Delta_c c^{\dagger}_{\mathbf{k}\uparrow} c^{\dagger}_{\text{-}\mathbf{k}\downarrow} + \Delta_f f^{\dagger}_{\mathbf{k}\uparrow} f^{\dagger}_{\text{-}\mathbf{k}\downarrow} + h.c. \right) \tag{14}$$

Here, we assume that in addition to the shift in the f-electron dispersion by $\pm IS$ arising from the magnetic Heisenberg exchange I between neighboring magnetic moments, the unscreened moment also couples to the light band via the Kondo coupling J. However, since the light states are shifted away from the Fermi energy due to the hybridization with the heavy f-electron states, the effect of the Zeeman splitting $\pm JS$ on the low energy density of states is negligible. Moreover, since $V \gg \Delta_{c,f}$ (reflecting the experimental observation that the hybridization gap is much larger than the superconducting gap), the low-energy density of states is predominantly determined by the f-electron states, explaining why the Hamiltonians in Eqs. (6) and (14) yield

essentially identical results for the low energy density of states. Finally, we note that Δ_f represents the effective superconducting gap for the f-electrons which is proximity-induced not only by the surface c-electrons, but also by the bulk c-electrons. The inclusion of bulk bands in the Hamiltonian of Eq. (13) is computational too demanding to be implemented here, thus requiring the implementation of the effective gap Δ_f (similar to the approach employed in Ref. [6]).

Supplementary Note 4. Experimental verification of spin polarization effects

Applying an external magnetic field can be an option to control the tip magnetization state and to detect the corresponding changes of the spin-polarized tunneling current. However, already a very small magnetic field can destroy the superconductivity of the Re(0001) substrate having a critical field of ~20 mT only. Alternatively, we managed to prepare a Ce-coated STM tip, which is oppositely magnetized to the one used for the measurements as described in the main part of the manuscript. As discussed together with Fig. S4(f), if the STM tip is spin-polarized, the measured dI/dV curve becomes asymmetric in energy. By reversing the spin polarization of the tip, the asymmetry in the dI/dV spectrum is expected to be inverted. This is indeed observed experimentally, as shown in Fig. S5.

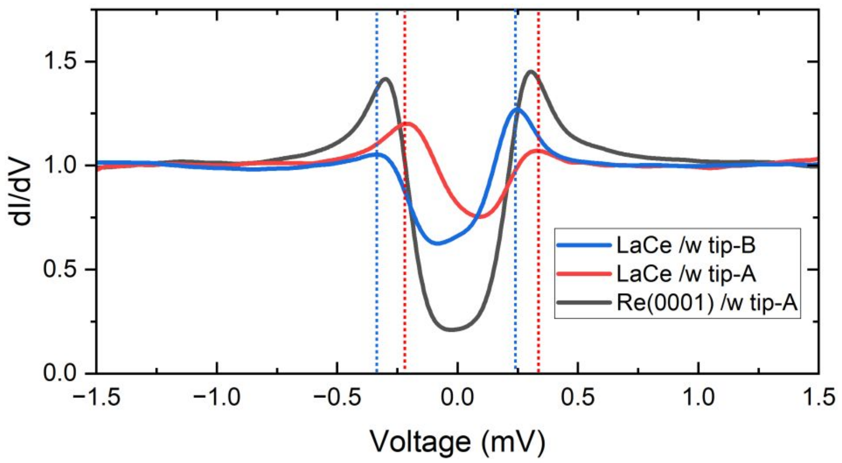

Supplementary Figure S5. Differential tunneling conductance (dI/dV) curves recorded before (tip-A, red) and after (tip-B, blue) the modification of the STM tip apex by a gentle indentation of the tip into the Re(0001) surface covered by Ce clusters as shown in Fig. 1(a). Both dI/dV

curves are obtained at the same location of the La-Ce layer. Red and blue dotted lines indicate the locations of maxima in the spectra.

Supplementary Note 5. Information about tunneling spectroscopic measurements

The two tunneling spectra presented in Fig. 3(a) were recorded at the same location. The tunneling spectra displayed in Fig. 3(b) are not the result of subtracting the background from the spectra in Fig. 3(a). Both spectra were obtained by independent tunneling spectroscopic measurements. Note that the spectra in Fig. 3(a) (or Fig. 4(b)) were recorded to simultaneously reveal the Kondo hybridization gap together with low-energy excitations inside the proximity-induced superconducting gap of the LaCe layer. Although one can see the signature of the proximity-induced gap in Fig. 3(a), the chosen measurement parameters lead to an instrumental broadening for the low energy excitations. Therefore, we took the spectra with different tunneling conditions (Fig. 3(b) or Fig. 4(c)) so that we can avoid the instrumental broadening as well as enhance the signal-to-noise ratio to access the low energy excitations. The stabilizing tunneling conditions for the spectra in Fig. 3(a) were: 1.0 nA and 15 mV, while for Fig. 3(b): 1.0 nA and 1.2 mV. The parameters for the lock-in detection of the differential tunneling conductance were: V_{mod} = 0.3 mV_{rms} and V_{mod} = 0.03 mV_{rms} for Fig. 3(a) and Fig. 3(b), respectively.

REFERENCES

(1) Maltseva, M.; Dzero, M.; Coleman, P. Electron Cotunneling into a Kondo Lattice. *Phys. Rev. Lett.* **2009**, *103* (20), 206402. https://doi.org/10.1103/PhysRevLett.103.206402.

(2) Morr, D. K. Theory of Scanning Tunneling Spectroscopy: From Kondo Impurities to Heavy Fermion Materials. *Rep Prog. Phys.* **2017**, *80* (1), 014502. https://doi.org/10.1088/0034-4885/80/1/014502.

(3) Coleman, P. New Approach to the Mixed-Valence Problem. *Phys. Rev. B* **1984**, *29* (6), 3035–3044. https://doi.org/10.1103/PhysRevB.29.3035.

(4) Newns, D. M.; Read, N. Mean-Field Theory of Intermediate Valence/Heavy Fermion Systems. *Adv. Phys.* **1987**, *36* (6), 799–849. https://doi.org/10.1080/00018738700101082.

(5) Kotliar, G.; Liu, J. Superexchange Mechanism and d -Wave Superconductivity. *Phys. Rev. B* **1988**, *38* (7), 5142–5145. https://doi.org/10.1103/PhysRevB.38.5142.

(6) Figgins, J.; Mattos, L. S.; Mar, W.; Chen, Y.-T.; Manoharan, H. C.; Morr, D. K. Quantum Engineered Kondo Lattices. *Nat. Commun.* **2019**, *10* (1), 5588. https://doi.org/10.1038/s41467-019-13446-1.

(7) Palacio-Morales, A.; Mascot, E.; Cocklin, S.; Kim, H.; Rachel, S.; Morr, D. K.; Wiesendanger, R. Atomic-Scale Interface Engineering of Majorana Edge Modes in a 2D Magnet-Superconductor Hybrid System. *Sci. Adv.* **2019**, *5* (7), eaav6600. https://doi.org/10.1126/sciadv.aav6600.
